# Genetics, Gene Flow, and Glaciation: The Case of the South American Limpet *Nacella mytilina*

**DOI:** 10.1371/journal.pone.0161963

**Published:** 2016-09-06

**Authors:** Claudio A. González-Wevar, Sebastián Rosenfeld, Nicolás I. Segovia, Mathias Hüne, Karin Gérard, Jaime Ojeda, Andrés Mansilla, Paul Brickle, Angie Díaz, Elie Poulin

**Affiliations:** 1 GAIA Antártica – Universidad de Magallanes, Departamento de Recursos Naturales, Bulnes 01890, Punta Arenas, Chile; 2 Instituto de Ecología y Biodiversidad (IEB), Departamento de Ciencias Ecológicas, Facultad de Ciencias, Universidad de Chile, Las Palmeras # 3425, Ñuñoa, Santiago, Chile; 3 Laboratorio de Macroalgas Antárticas y Subantárticas, Universidad de Magallanes, casilla 113-D, Punta Arenas, Chile; 4 Fundación Ictiológica, Providencia – Santiago, Chile; 5 South Atlantic Environmental Research Institute (SAERI), PO Box 609, Stanley Cottage, Stanley, Falkland Islands; 6 Departamento de Zoología, Facultad de Ciencias Naturales y Oceanográficas, Universidad de Concepción, Concepción, Chile; University of California, UNITED STATES

## Abstract

Glacial episodes of the Quaternary, and particularly the Last Glacial Maximum (LGM) drastically altered the distribution of the Southern-Hemisphere biota, principally at higher latitudes. The irregular coastline of Patagonia expanding for more than 84.000 km constitutes a remarkable area to evaluate the effect of Quaternary landscape and seascape shifts over the demography of near-shore marine benthic organisms. Few studies describing the biogeographic responses of marine species to the LGM have been conducted in Patagonia, but existing data from coastal marine species have demonstrated marked genetic signatures of post-LGM recolonization and expansion. The kelp-dweller limpet *Nacella mytilina* is broadly distributed along the southern tip of South America and at the Falkland/Malvinas Islands. Considering its distribution, abundance, and narrow bathymetry, *N*. *mytilina* represents an appropriate model to infer how historical and contemporary processes affected the distribution of intraspecific genetic diversity and structure along the southern tip of South America. At the same time, it will be possible to determine how life history traits and the ecology of the species are responsible for the current pattern of gene flow and connectivity across the study area. We conducted phylogeographic and demographic inference analyses in *N*. *mytilina* from 12 localities along Pacific Patagonia (PP) and one population from the Falkland/Malvinas Islands (FI). Analyses of the mitochondrial gene COI in 300 individuals of *N*. *mytilina* revealed low levels of genetic polymorphism and the absence of genetic differentiation along PP. In contrast, FI showed a strong and significant differentiation from Pacific Patagonian populations. Higher levels of genetic diversity were also recorded in the FI population, together with a more expanded genealogy supporting the hypothesis of glacial persistence of the species in these islands. Haplotype genealogy, and mismatch analyses in the FI population recognized an older and more complex demographic history than in PP. Demographic reconstructions along PP suggest a post-LGM expansion process (7.5 ka), also supported by neutrality tests, mismatch distribution and maximum parsimony haplotype genealogies. Migration rate estimations showed evidence of asymmetrical gene flow from PP to FI. The absence of genetic differentiation, the presence of a single dominant haplotype, high estimated migration rates, and marked signal of recent demographic growth, support the hypothesis of rapid post-glacial expansion in *N*. *mytilina* along PP. This expansion could have been sustained by larval and rafting-mediated dispersal of adults from northernmost populations following the Cape Horn Current System. Marked genetic differentiation between PP and FI could be explained through differences in their respective glacial histories. During the LGM, Pacific Patagonia (PP) was almost fully covered by the Patagonian Ice Sheet, while sheet coverage in the FI ice was restricted to small cirques and valleys. As previously recorded in the sister-species *N*. *magellanica*, the FI rather than represent a classical glacial refugium for *N*. *mytilina*, seems to represent a sink area and/or a secondary contact zone. Accordingly, historical and contemporary processes, contrasting glacial histories between the analyzed sectors, as well as life history traits constitute the main factors explaining the current biogeographical patterns of most shallow Patagonian marine benthic organisms.

## Introduction

The development of large continental ice sheets during glacial episodes of the Quaternary, most recently the last glacial maximum (LGM), between 23 and 18 ka, generated major climate and environmental changes that deeply affected the distribution of the present day biota [1–3]. Several studies have demonstrated how these episodes radically altered the demography and the geographical range of species and populations and therefore also affected the distribution of intraspecific genetic variation [1,3–7]. A vast array of records from the Northern Hemisphere provided the empirical basis for the Expansion-Contraction (E-C) model of Pleistocene biogeography [1], describing the response of populations and species to climatic change [2–4,8–11]. Under a simple E-C model, cool-temperate species survived ice advances at lower latitude refugia, only re-populating higher latitudes through range expansion [1,2,5,12]. This model constitutes a fundamental paradigm of Quaternary biogeography and also provides a potentially useful explanation to understand how species assemblages will respond to future climate change [3]. The development of molecular-based techniques for population genetic and phylogeographic studies has revolutionized our understanding of the consequences of past glacial processes concerning patterns of diversity, connectivity and structure at different geographical and temporal scales [2,4,5,10,13]. The Quaternary history of South America is fairly well understood with glacial events generating major changes in sea level, climate, and landscape [14–17]. During the LGM most of the Pacific Patagonian (PP) fjords and channels from Chiloé Island (42°S) to Cape Horn (56°S) were covered by the Patagonian Ice Sheet, expanding more than 480.000 km^2^ with an ice volume of around 500.000 km^3^ [14,15,18,19].

During the last three decades more and more genetic data have been accumulated for different groups of Patagonian taxa. They include population-based and phylogeographic studies in aquatic invertebrates [20–25], freshwater [26–31] and marine [32–35] fishes, lizards [36,37], amphibians [38], mammals [39–43], and plants [44–47]. Most of these studies evidenced clear patterns of postglacial recolonization and recognized the presence of Quaternary glacial refugia on the East side of the Andes, along the Patagonian Steppe [26,29], with refugia on the West side of Andes both within [23,29,38] and outside of the glacier limits [26,34]. Absence of genetic structure and a strong signal of recent demographic growth recorded in shallow Patagonian marine benthic invertebrates support the hypothesis of rapid postglacial expansion [20–22,25]. Species with high dispersive potential (i.e., *Durvillaea antarctica* and *Macrocystis pyrifera*) recolonized Patagonia since the LGM from geographically distant regions [46,48] evidence of the major role of long-distance dispersal in the biogeography of Subantarctic near-shore marine benthic organisms [49–51].

True limpets of the genus *Nacella* (Patellogastropoda: Nacellidae) are dominant organisms of Antarctic and Subantarctic inter- and sub-tidal rocky ecosystems. As typical patellogastropods, the members of *Nacella* are dioecious organisms with external fertilization and in the Antarctic limpet *Nacella concinna* the larval lifespan can extend for more than two months [52–54]. The genus includes at least 11 nominal species currently distributed in different provinces of the Southern Ocean inclunding maritime Antarctica, South America, and Subantarctic islands (South Georgia, South Sandwich, Marion, Kerguelen, Heard, Macquarie, and Campbell) [55]. Recent molecular studies have helped to further understand the biogeography of the genus across its distribution in the Southern Ocean [56], and particularly in Patagonia [20,24,25]. Geographically distant species from the Antarctic Peninsula (*Nacella concinna*), central Chile (*Nacella clypeater*), Patagonia (*Nacella magellanica*, *Nacella deaurata*, *Nacella flammea*, and *Nacella mytilina*) and Heard Island (*Nacella kerguelenensis* and *Nacella* cf. *macquariensis*) showed high levels of genetic divergence indicating the existence of transoceanic discontinuities in the genus [56]. At the same time, divergence time estimations indicate that the origin and diversification of *Nacella* is relatively modern and not associated with continental drift processes but with climatic and oceanographic processes associated to the middle Miocene Climatic Transition (< 15 Ma) [56]. South America, and particularly Patagonia exhibits the highest taxonomic richness, with at least eight nominal species [55,57]. Hence, this region was considered as the center of origin of the genus from where seaweed-associated species spread eastward to other Subantarctic areas [55]. Nonetheless, recent molecular studies and divergence time estimations indicate that number of Patagonian species of *Nacella* was overestimated and that they constitute a recently derived South-American lineage [20,24,56]. Moreover, recent molecular and morphological analyses indicate that the recent radiation of *Nacella* in Patagonia includes only four evolutionary significant units: *N*. *deaurata*, *N*. *flammea*, *N*. *magellanica* and *N*. *mytilina* [20,24].

One of the Patagonian *Nacella* species, the kelp-dweller *Nacella mytilina* (Helbling, 1779), exhibits the broadest distribution of the genus including the southern tip of South America, the Falkland/Malvinas Islands, and Kerguelen Island, located more than 7000 kilometers away [55]. Nevertheless, recent molecular studies indicate that *N*. *mytilina*-like individuals from Kerguelen Island constitute a particular kelp-associated morphotype of *N*. *kerguelenensis* (González-Wevar, under review). Kelp-associated individuals from Kerguelen Island and Patagonian *N*. *mytilina* showed more than 8.04% of genetic divergence, similar to the degree of divergence detected between South American and Antarctic species of the genus [56]. Accordingly, *N*. *mytilina* is restricted to the southern tip of South America and exhibits a narrower distribution than other Patagonian relatives (*N*. *magellanica* and *N*. *deaurata*). The species is currently found between Guarelo Island (50° S) in the Pacific to the Santa Cruz Province (45°–52°S) in the Atlantic, including the Strait of Magellan, Cape Horn, Tierra del Fuego, and the Falkland/Malvinas Islands [25,55–57]. The species is relatively abundant over macroalgae such as *Macrocystis pyrifera*, *Gigartina skottsbergii*, and *Lessonia* [58,59]. Considering its habitat preferences, *N*. *mytilina* is morphologically well distinguished from other Patagonian limpets in terms of shell shape, sculpture and thickness. Recent comparative dietary composition analyses in Patagonian *Nacella* species indicate that *N*. *mytilina*’s diet is highly specialized compared to its relatives *N*. *magellanica* and *N*. *deaurata*. *Nacella mytilina* feeds over *M*. *pyrifera* and particular kelp-associated diatoms [60]. At the same time, all Patagonian *Nacella* species, with the exception of *N*. *mytilina*, showed changes in their dietary composition between winter and summer [60]. Recent phylogeographic studies of its relatives *N*. *magellanica* and *N*. *deaurata*, along the Atlantic coast recorded medium levels of genetic diversity, an absence of genetic structure, and evidence of recent demographic expansions [20]. Similarly, a study conducted in *N*. *magellanica* along PP also registered an absence of genetic structure, the presence of a single dominant haplotype, high levels of gene flow, and strong signals of recent demographic expansion [25]. Nevertheless, in the same study González-Wevar et al. [25] recorded significant differences between Pacific Patagonia (PP) and Falkland/Malvinas Islands (FI) populations that could be a consequence of their distinct glacial histories. The current knowledge about the pattern of genetic diversity in *N*. *mytilina* is restricted to a single locality in the Strait of Magellan [24]. Therefore, phylogeographic analyses are required in order to further understand the legacy of the glacial Quaternary cycles over this kelp-associated species, as well as the role of rafting in the patterns of genetic connectivity.

## Methods

### Ethics Statement

This work was conducted using “mauchos” (*Nacella mytilina*) as study model, a common limpet species from the southern tip of South America. The species is not protected by the Chilean Fishery Subsecretary and has not been included in the Chilean fishery statistics. Permission to undertake field studies and to collect specimens was issued by the Chilean Fishery Service Director (Carlos Orellana Céspedes), under the technical memorandum (249/2015). Permits to undertake field studies and collect specimens in Falkland Islands were issued by the Environmental Planning Department, the Falkland Islands Government. The Instituto de Ecología y Biodiversidad (IEB/15-2015) and Chilean Fishery Service (SERNAPESCA 429/2015) ethic committees approved sampling protocols and experiments. For this, we complied with local legislation and the Convention on Biological Diversity.

### Samplings, DNA preparation, PCR amplification and sequencing

**Fig 1:**
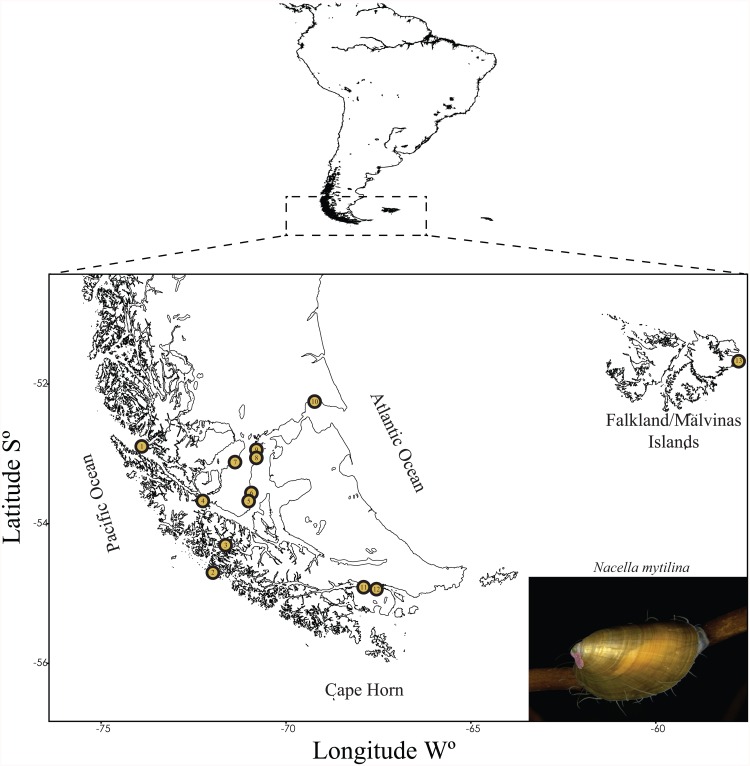
Sampling localities of *N*. *mytilina* along Pacific Patagonia (PP) and the Falkland/Malvinas Islands (FI). Shapefiles of the Patagonian coastlines available in the database of GEOdas (NOAA) and were filtered using GEOdas Coastline Extractor v. 1.1.3.1 (https://www.ngdc.noaa.gov/mgg/geodas/geodas.html). 1) Tamar Island (PP); 2) London Island (PP); 3) Duntze Sound (SM); 4) Carlos III Island (SM), 5) Santa Ana Point (SM), 6) Carrera Point (SM), 7) Otway Sound (SM), 8) Chabunco (SM), 9) Laredo Bay (SM), 10) Possession Bay (SM), 11) Virginia Bay (CH), 12) Paula Bay (CH) and 13) Hookers Point. SM = Strait of Magellan, PP = Pacific Patagonia; CH = Cape Horn; FI = Falkland/Malvinas Islands. Photograph of *N*. *mytilina* over *Macrocytis pyrifera* courtesy of César Cárdenas (ccardenas@inach.cl).

Individuals were sampled from 13 localities along Pacific Patagonia (PP) and the Falkland/Malvinas Islands (FI; Fig 1). Samples from PP populations (n = 12) were collected along the southern Pacific coast of Patagonia, the Strait of Magellan and Cape Horn and included: 1) Tamar Island (52° 54’ S; 73° 48’ W), 2) Duntze Sound (54°19’S; 73°48’W), 3) London Island (54°40’S; 72°00’W), 4) Carlos III Island (53°39’S; 72°23’W), 5) Santa Ana Point (53°37’S; 70°54’W), 6) Carrera Point (53°35’S; 70°55’W), 7) Otway Sound (53°06’S; 71°20’W), 8) Chabunco (52°59’S; 70°48’W), 9) Laredo Bay (52°56’S; 70°47’W), 10) Possession Bay (52°13’S; 69°17’W), 11) Virginia Bay (54°54’S; 67°36’W) and 12) Paula Bay (54°56’S; 67°39’W; Fig 1). Individuals from the Falkland/Malvinas Islands (FI) were collected at Hookers Point (51°42’S; 57°46’W; Fig 1).

Whole specimens were fixed in ethanol (99%) and DNA was prepared from the mantle using a salting-out methodology [61]. A partial fragment of the mitochondrial gene Cytochrome c Oxidase Subunit I (COI) was amplified using universal primers [62]. PCR amplifications were performed in a 25 μl reaction containing 2.5 μl 10X Buffer (50 mM KCl, 10 mM Tris-HCl, pH 8.0), 1.0 μl of 50 mM MgCl_2_, 200 mM dNTPs, 0.5 μl of each primer (10 pg/μl), 1 U Taq polymerase (Invitrogen), 16.7 μl of double-distilled water and 50 ng of DNA. Thermal cycling parameters included an initial denaturation step at 94°C for 5 min, followed by 35 cycles at 94°C (60 sec), 48°C (60 sec), 72°C (90 sec), and a final 5 min extension at 72°C. PCR amplicons were purified using QIAquick Gel Extraction Kit (QIAGEN) and sequenced in both directions with an Automatic Sequencer ABI3730 x 1 at Macrogen Inc. (Seoul, South Korea).

### Genetic diversity and population structure in *N*. *mytilina*

The COI sequences of *Nacella mytilina* were edited using Proseq v. 3.5 [63], and aligned with ClustalW [64]. New COI sequences in *N*. *mytilina* were added to previous data for the species [24] and have been deposited in Genbank under the Accession Numbers KX600474—KX600492. A DNA saturation analysis following Xia and Xie [65] was performed to evaluate how saturation of transitions accumulates in relation to nucleotide divergence in the complete *N*. *mytilina* COI data set. Levels of genetic polymorphism were estimated using standard diversity indices, such as the number of haplotypes (*k*), the number of segregating sites (*S*), haplotypic diversity (*H*), the average number of pairwise differences (*II*), and nucleotide diversity (*π*) for each locality, and for the complete COI data set using DnaSP v.5.00.07 [66]. Levels of genetic differentiation between the analyzed localities were estimated following Pons & Petit [67] through main pairwise differences (N_ST_) and haplotype frequencies (G_ST_) in Arlequin v. 3.5 [68]. The statistical significance of genetic pairwise differences was determined using permutation tests (20 000 iterations) of haplotype identities. Similarly, we estimated the levels of genetic differentiation using the nearest-neighbor statistic (S_nn_) that measure how often nearest neighbor’s (in sequence space) sequences are from the same locality in geographic space [69]. The statistical significance of S_nn_ analyses was determined through a permutation test using 20 000 iterations.

Two different clustering methods were used to determine the spatial genetic structure of *N*. *mytilina*. First, we estimated the number and the composition of panmictic groups and the spatial boundaries among them using a Bayesian model computed in GENELAND v. 2.0.0 [70] in the R environment (R, version 2.4.1; [71]. This R package implements a Markov Chain Monte Carlo (MCMC) procedure to determine the best clustering of samples with regard to genetic and geographic information. The geographic information is considered at the Bayesian prior level, so that clusters corresponding to spatially structured groups are considered to be more likely than clusters that are randomly distributed in space. Analyses were run using 50 x 10^6^ MCMC iterations sampled each 1000 steps. Assembled MCMC scores were graphed against generations using Tracer v.1.5 (http://beast.bio.ed.ac.uk/Tracer) to identify stationarity, and therefore determine the number of generations that must be discarded as burn-in. In the particular case of *N*. *mytilina* a 10% of the trees were discarded as burn-in. A maximum number of clusters (K = 14) were run to estimate the model parameters and posterior probabilities of group membership. Second, we determined the spatial genetic structure in *N*. *mytilina* using Arlequin [68] by estimating the number and composition of groups that were most differentiated based on sequence data with Analysis of Molecular Variance (AMOVA). This analysis uses multiple spatial scales in statistical methods to characterize spatial genetic structure by partitioning the variance: within populations, among populations within groups, and among groups. Finally, we performed a test for isolation by distance using a Mantel test with 10 000 permutations in Arlequin to determine the correlation between Slatkin’s linearized localities genetic differentiation [72] and the linear geographic distance (km) between populations measured using FOSSIL [73].

### Demographic inference in *N*. *mytilina*

Genealogical relationships in *N*. *mytilina* were constructed using parsimony networks in Hapview (http://www.cibiv.at/~greg/haploviewer). At the same time, we performed neutrality statistical tests (Tajima’s D and Fu’s F_S_) using DnaSP for the whole COI data set and for each recognized group to estimate whether sequences deviate from mutation-drift equilibrium. At the same time, population demographic histories were estimated comparing the distribution of pairwise differences between haplotypes (mismatch distribution) for each recognized group to the expected distribution under the sudden expansion growth model of Rogers & Harpending [74]. This analysis is a common phylogeographic method since the amount of nucleotide differences between haplotypes depends on the length of time since they diverged. This analysis rests in the estimation of three parameters: i) τ = the date of growth/decline measured in units of mutational time (τ = 2μt) where t = time in years and μ = mutational rate per sequence per year, ii) initial theta *θ*_*i*_ = *2 N*_*i*_*μ* before the population growth/decline) and iii) final theta *θ*_*t*_ = *2 N*_*t*_*μ* after population growth/decline. The demographic expansion parameters were estimated using the nonlinear least square approach described by Schneider & Excoffier [75] implemented in Arlequin. The goodness of fit between the observed and expected mismatch distributions was tested using a parametric bootstrap approach that uses the sum of squared deviations as a statistic test implemented in Arlequin.

### Gene flow and connectivity

Different models of gene flow were compared between the recorded groups in *N*. *mytilina* (PP and FI) to test for different scenarios using the software MIGRATE v.3.5 [76]. For this purpose, we defined four candidate models constraining the existence of directionality of gene flow between the recorded genetic groups. The first model allowed bidirectional gene flow between PP and FI (full island model). Asymmetric levels of gene flow between the recorded groups defined models 2 (from PP to FI) and 3 (from FI to PP). The last model assumed PP and FI as the same panmictic population. All the analyses were run using a HKY + I substitution model and transition-transversion ratio of 9.9401 as previously estimated by jModeltest v2 [77]. The specific substitution rate for the selected marker was set to constant, as suggested by the developer. Analysis consisted of one long chain with 500 000 recorded parameter steps, a sampling interval of 100 and a burn-in of 10%, running multiplate replicates (10 independent chains). A heated scheme (1.0, 1.5, 3.0. and 1000000.0) was used to calculate the marginal likelihoods for model comparisons. We used a thermodynamic integration approximation (T.I.) for the log-equivalent Bayes Factor (LBF) because this analysis results in LBFs with high repeatability and little variance [78]. Higher T.I. values indicate a better fit of the model than lower ones. Finally, the associated probability of each model in relation to other was estimated following Kaas and Rafery [79].

Finally, we estimated different demographic indices and evaluated two asymmetric isolation-with-migration models between Pacific Patagonia (PP) and the Falkland/Malvinas Islands (FI) using IMa2 software [80,81]. The first model: from Pacific Patagonia to the Falkland/Malvinas Island (m PP > FI) and the second one: from the Falkland/Malvinas Islands to Patagonia (m FI > PP). We carried out separately several preliminary runs in the M-mode (Markov Chain Monte Carlo; MCMC mode) of the software to determine the best set of priors that ensure mixing and convergence. Uniform priors were used to determine the effective population size (Θ1, Θ2, and ancestral Θa, Θ = 600) and splitting time (t = 30), whereas an exponential prior (mean = 20) for gene flow (m) was adopted. We performed 80 x 10^6^ MCMC steps sampling every 100 generations, with a burn-in period of 10%. COI sequences in *N*. *mytilina* were assumed to mutate under the HKY mutation model following Hey and Nielsen [81]. Once convergence was achieved under the M-mode, we used the same simulated genealogies under the L-Mode (Load Tree mode) to determine the log maximum-likelihood and credibility intervals (95% under HPD) estimates for migration parameters using a likelihood ratio test. Under the L-Mode we compared our data with a null model of no migration [80,81].

## Results

### Genetic diversity and structure in *N. mytilina*

The complete COI data set in *N*. *mytilina* included 300 individuals and consisted of 687 nucleotide positions coding for 229 aminoacids. As expected analyzing coding regions, no insertion/deletion or stop codons were detected in the whole data set. Sequences were not saturated at any position and a single amino acid substitution (from Alanine to Valine at amino acid 182) was detected using the invertebrate mitochondrial table [82]. Low levels of genetic variability characterized *N*. *mytilina*, with 26 polymorphic characters (3.78%); 16 of them (61.53%) were parsimoniously informative. As previously estimated in other species of *Nacella* [24,25,56], sequences were A-T rich (60.8%) compared to the mean G-C content. Haplotype diversity (*H*) varied between 0.284 (Carlos III Island) and 0.822 (Hookers Point, FI; Table 1). The number of polymorphic sites (*S*) varied between 3 (several PP localities; Table 1) and 19 (Hookers Point, FI). Similarly, the number of haplotypes (*k*) varied between 4 (several PP localities) and 8 (Hookers Point and Laredo Bay; Table 1). The average number of nucleotide differences (*II*) and mean nucleotide diversity (*π*) were low in most localities of PP, while the diversity of these indices was comparatively higher in FI (Table 1). In fact, permutation test analyses (100,000 iterations) detected significant higher values for *II* (P = 0.00053) and *π* (0.00048) in the FI population compared to those from PP (Table 1). Finally, the number of private haplotypes (6/8) was higher in FI than in any PP locality (Table 1).

**Table 1:** Diversity indices and neutrality tests in *Nacella mytilina* along Pacific Patagonia and the Falkland/Malvinas Islands.

Locality	*n*	*k*	*H*	*S*	*II*	*π*	p.a.	Tajima´s D	Fu´s FS
Tamar Island	21	4	0419	3	0.457	0.0006	0	-1.18	-1.72
Duntze Sound	18	4	0.608	3	0.843	0.0012	0	-0.09	-0.50
London Island	25	4	0.357	4	0.600	0.0008	0	-1.17	-0.95
Carlos III Island	20	4	0.284	4	0.489	0.0007	0	-1.63*	-1.61
Carrera Bay	18	5	0.405	5	0.654	0.0009	0	-1.74*	-2.38
Santa Ana	30	4	0.409	4	0.549	0.0008	0	-1.17	-2.24
Otway Sound	18	4	0.314	3	0.314	0.0004	2	-1.71	-2.60
Chabunco	22	5	0.528	3	0.667	0.0009	1	-0.49	-2.60
Laredo Bay	28	8	0.542	8	0.765	0.0011	2	-1.93*	-5.37*
Possession Bay	23	5	0.391	5	0.585	0.0008	0	-1.66	-2.36
Virginia Bay	26	4	0.345	3	0.425	0.0006	0	-1.12	-1.70
Paula Bay	27	7	0.627	6	0.838	0.0012	2	-1.35	-3.59*
PP Total	276	13	0.435	12	0.599	0.0008	-	-1.62*	-9.64*
Hookers Point	24	8	0.822	19	4.18	0.0060	6	-0.64	0.68
Total	300	19	0.481	26	0.966	0.0014		-2.11**	-13.49***

Where: *n* = number of analyzed individuals; *k* = number of haplotypes; *S* = polymorphic sites; *H* = haplotype diversity; *II* = average number of pairwise differences; *π* = nucleotide diversity; p.a. = private haplotypes.

* p<0.05,

** p<0.01,

*** p<0.001.

Mean general values of differentiation measured over 13 populations of *N*. *mytilina* were low, especially considering average G_ST_ = 0.025 and N_ST_ = 0.032. At the same time, pairwise comparisons based on N_ST_ and G_ST_ did not recognize significant structure among PP populations of *N*. *mytilina* from the Strait of Magellan to Cape Horn (Table 2). In contrast, G_ST_ and N_ST_ pairwise comparisons detected significant differences between the FI population and the rest of the PP ones (Table 2). Considering the pattern of genetic structure recorded in the species, we examined the significance of the correlation between genetic divergence measured as Slatkin’s linearized F_ST_ [Phi_ST_/(1- Phi_ST_)] and geographical distance among PP localities using a Mantel test implemented in Arlequin. The associated probabilities of these analyses were estimated using 25 000 permutations. We didn’t detect any significant correlation between genetic and geographic distance along PP (r = 0.001; P = 0.6).

**Table 2:** Pairwise G_ST_ (below the diagonal) and N_ST_ (above the diagonal) values calculated among the analyzed populations of *Nacella mytilina*. 20 000 iterations. Statistical significant differences are marked in bold.

	**1**	**2**	**3**	**4**	**5**	**6**	**7**	**8**	**9**	**10**	**11**	**12**	**13**
**1**	----	0.023	0.000	0.000	0.000	0.000	0.000	0.000	0.000	0.000	0.000	0.014	**0.193**
**2**	0.000	----	0.000	0.026	0.000	0.000	0.074	0.000	0.008	0.013	0.000	0.000	**0.131**
**3**	0.000	0.027	----	0.000	0.000	0.000	0.009	0.000	0.000	0.000	0.000	0.000	**0.202**
**4**	0.000	0.047	0.000	----	0.000	0.000	0.000	0.000	0.000	0.000	0.000	0.009	**0.192**
**5**	0.000	0.000	0.000	0.000	----	0.000	0.000	0.000	0.000	0.000	0.000	0.000	**0.175**
**6**	0.000	0.000	0.000	0.000	0.000	----	0.009	0.000	0.000	0.000	0.000	0.000	**0.204**
**7**	0.000	0.000	0.000	0.000	0.000	0.000	----	0.025	0.000	0.000	0.001	0.043	**0.201**
**8**	0.000	0.000	0.003	0.016	0.000	0.000	0.010	----	0.000	0.000	0.000	0.000	**0.186**
**9**	0.000	0.000	0.000	0.021	0.000	0.000	0.004	0.000	----	0.000	0.000	0.005	**0.206**
**10**	0.000	0.000	0.000	0.000	0.000	0.000	0.000	0.000	0.000	----	0.000	0.000	**0.191**
**11**	0.000	0.000	0.000	0.000	0.000	0.000	0.000	0.000	0.000	0.000	----	0.000	**0.200**
**12**	0.000	0.000	0.040	0.050	0.008	0.004	0.043	0.000	0.000	0.010	0.019	----	**0.159**
**13**	**0.141**	**0.084**	**0.197**	**0.206**	**0.148**	**0.156**	**0.186**	**0.102**	**0.103**	**0.156**	**0.177**	**0.052**	----

**Fig 2:**
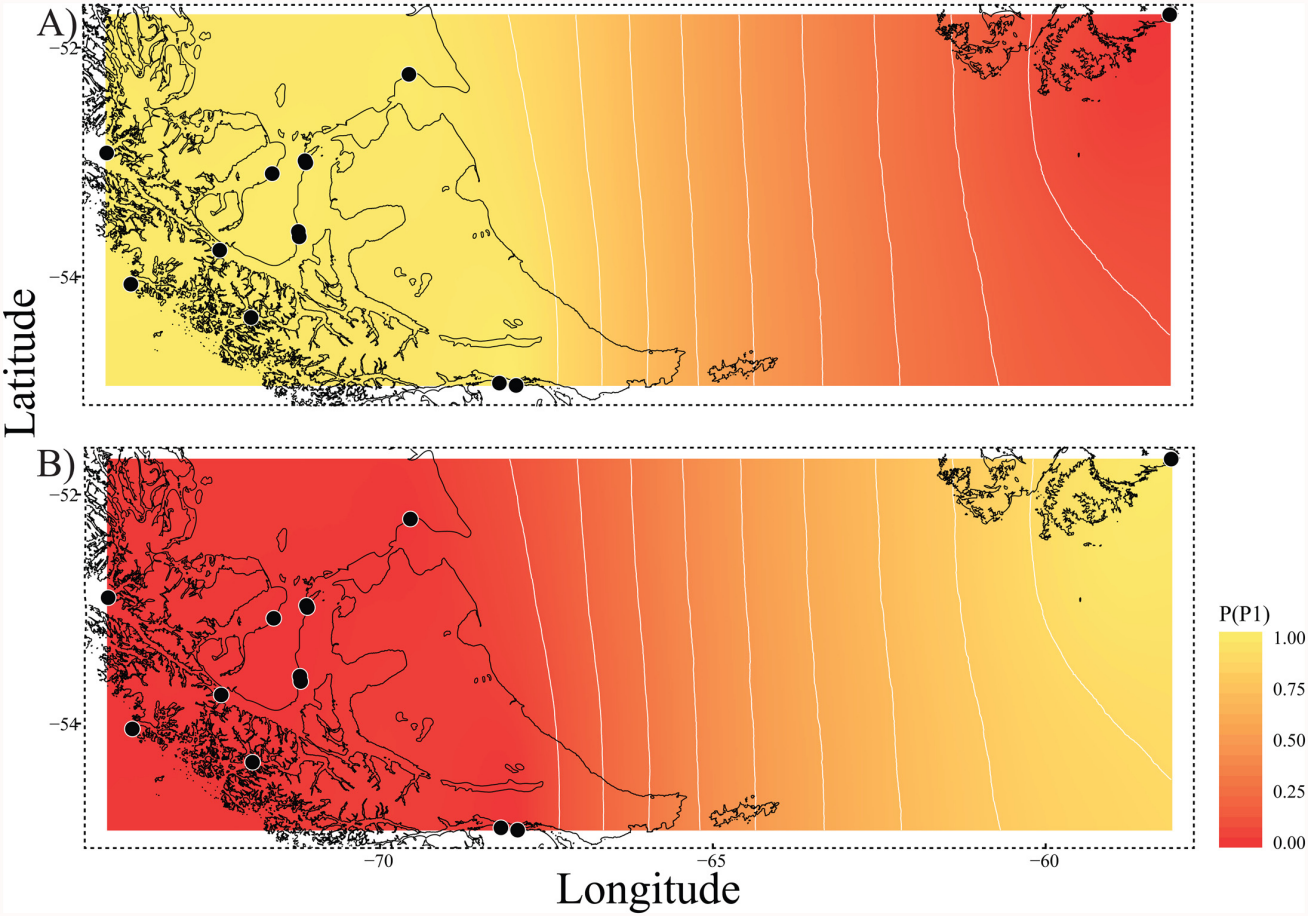
Spatial output from Geneland using all 13 *N*. *mytilina* populations. Black circles indicate the relative position of the sampling localities. Darker and lighter shading are proportional to posterior probabilities of membership to clusters, with lighter (yellow) areas showing the highest probabilities of clusters. Posterior probabilities of membership were plotted to the *shapefiles* of the Patagonian coastline available in the database of GEOdas (NOAA) and filtered using GEOdas Coastline Extractor v 1.1.3.1 (https://www.ngdc.noaa.gov/mgg/geodas/geodas.html).

The nearest-neighbor statistic (S_nn_) in *N*. *mytilina* (S_nn_ = 0.09) showed low but significant levels of phylogeographic signal (P < 0.00001) for the whole COI data set. However, when this analysis was performed considering the pattern of genetic structure recorded in the species (PP vs FI) S_nn_ became very high (Snn = 0.9) showing the elevated degree of phylogeographic signal recorded between these two areas. This pattern of genetic structure was further supported by the model based on Bayesian clustering algorithm, which detected two main clusters (K = 2). The first cluster includes all the PP localities from the Strait of Magellan to Cape Horn (Fig 2A) while the second one included the individuals from FI (Fig 2B). Values of cluster membership were high for all localities (c.a. P = 0.9). The mean probability value (P = 0.5) corresponds to the boundary between the clusters run between Pacific Patagonia and the Falkland/Malvinas Islands (Fig 2). Similarly, AMOVA analyses detected two maximally differentiated groups accounting for 43.09% of the total variance, and only 1.17% was due to within-group variation among PP localities (Table 3). These groups were 1) Hookers Point, FI, and 2) the rest of the localities from Pacific Patagonia.

**Table 3:** Analysis of Molecular Variance (AMOVA) depicting the percentage of variation explained among groups (Pacific Patagonia and Falkland/Malvinas Islands), among populations within groups, and within populations. F_SC_: differentiation within populations among groups; F_CT_: differentiation among groups (** p<0.01, *** p<0.001).

Source of variation	d.f.	Sum of squares	Variance components	Percentage of variation
Among groups	1	14.645	0.32492 Va	43.09
Among populations within groups	11	2.455	0.00881 Vb	1.17
Within populations	305	133.567	0.43792 Vc	58.08
Total	317	150.667	0.75404	

Fixation Index

F_SC_: 0.02052 ***

F_CT_: 0.43091 ***

### Demographic inference in *N*. *mytilina*

**Fig 3:**
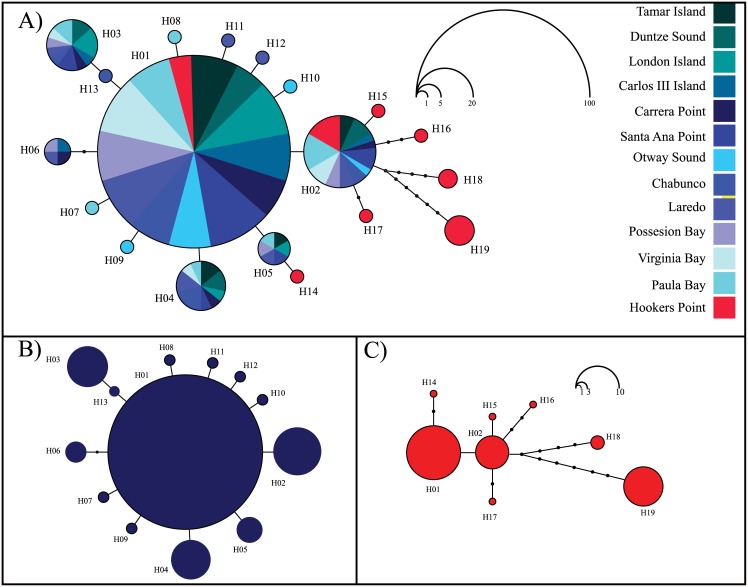
A) Parsimony haplotype network including 300 individuals of *Nacella mytilina* mtDNA COI sequences from 13 localities along PP and FI. Each haplotype is represented by a colored circle indicating the locality where it was collected. Circles sizes are proportional to the frequency of the haplotype in the whole sampling effort. B) General maximum parsimony haplotype network including PP populations. C) General maximum parsimony network in Hookers Point, FI. The size of the haplotypes circles (B and C) is proportional to the their frequency.

**Fig 4:**
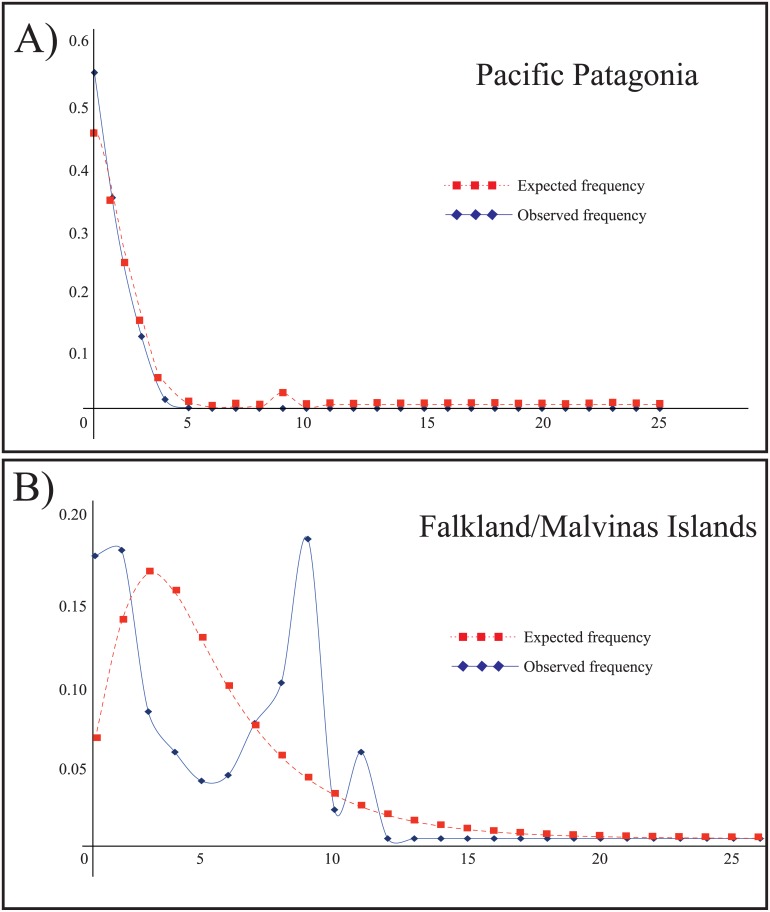
Pairwise difference distribution for the COI gene in *N*. *mytilina* populations from A) Pacific Patagonia (PP) and B) Falkland/Malvinas Islands (FI). X-axis = Pairwise differences and y-axis = frequency.

Parsimony network of *N*. *mytilina* recorded a total of 19 different haplotypes and showed a typical star-like topology and a very short genealogy (Fig 3A). A central haplotype (H01) was the most frequent one (71.33%) and distributed at all localities (Fig 3A). Following Posada and Crandall [83], this dominant haplotype should represent the most ancestral one, whereas the most derived one is related to it with a maximum branch length of 8 mutational steps (H19 from FI). A second haplotype (H02) of intermediate frequency (10%), separated by a single mutational step from H01, was also recorded in all the analyzed localities along PP and FI (Fig 3A). Four haplotypes (H03, H04, H05, and H06) were recorded in more than three individuals belonging to different PP localities (Fig 3A). A total of 10 singletons were recorded in the whole data set, six of them were recorded in PP and the rest in FI (Fig 3). Despite the high frequency of H01 in PP (>70%), that haplotype was present in only 33% of the sampled individuals from FI. Several haplotypes from FI (H14, H15, H16, and H17) were closely related to and even shared (H01 and H02) with the Patagonian diversity. Finally, two haplotypes of medium frequency recorded in FI (H18 and H19) are endemic to these islands and separated from H01 by five (H18) and seven (H19) mutational steps (Fig 3A). As expected for star-like genealogies, global Tajima’s D and Fu’s *F* neutrality tests were both negative and significant for the whole COI data set (Table 1). However, these neutrality tests showed contrasting results between the recorded genetic groups (PP and FI). Across PP, Tajima’s D and Fu’s F were negative and significant while both indices were not significant in FI (Table 1). Similarly, the distribution of pairwise differences varied considerable among the recognized genetic groups in *N*. *mytilina*. The mismatch distribution in PP was L-shaped (Fig 4A) and in FI it had a multimodal distribution (Fig 4B).

Sudden growth model analyses based on a 10% mutational rate detected that population expansion in PP occurred approximately 7.5 ka. The presence of a multimodal mismatch distribution in FI (Fig 4B), evidenced by the haplotype genealogy of this population and the absence of good-fit between the observed and expected distribution precluded the analysis of population expansion in this locality.

### Gene flow and connectivity

**Fig 5:**
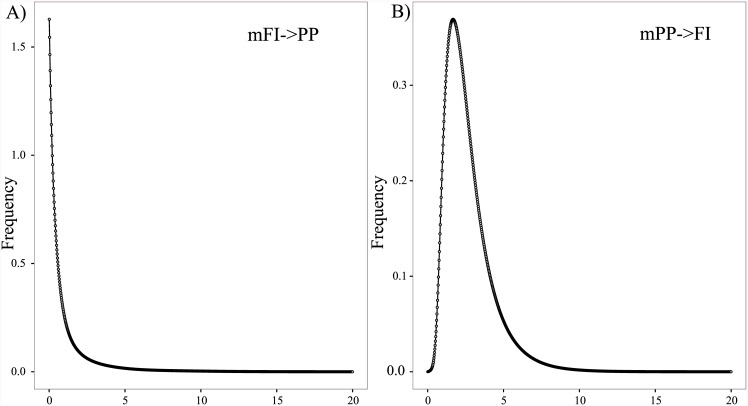
Marginal posterior probability distribution (frequency) of migration rate estimates in each direction for *N*. *mytilina* using IMa2. a) Migration pattern estimations from Falkland/Malvinas Islands (FI) to Pacific Patagonia (PP). B) Migration pattern estimations from PP to FI.

Gene flow analyses using MIGRATE and different migration models detected evidence of asymmetrical gene flow from PP to FI. Among the tested models the one from PP to FI received the highest probability (Table 4). Similarly, the coalescent approach of isolation-with-migration implemented in IMa2 detected overall medium levels of gene flow between PP and FI (Table 5). The model predicted a most likely pattern of asymmetric migration with highly significant migration from PP to FI (LRT, 40.723, Fig 5A), and no migration between FI to PP (LTR, 0.000, Fig 5B).

**Table 4:** Thermodynamic integration (T.I.) and log Bayes factor (LBF) comparisons for different migration models between Pacific Patagonia (PP) and the Falkland/Malvinas Islands (FI) in *Nacella mytilina*.

Model	T.I.	LBF	Model prob	Model rank
1. full migration	-1514.834	-30.194	<0.001	2
2. PP to FI	-1499.737	0	1	1
3. FI to PP	-1515.015	-30.555	<0.001	3
4. panmixia	-1545.644	-91.813	<0.001	4

**Table 5:** Estimates of migrations rates (m) in each direction and genetic diversities (Θ), based on the isolation-with-migration model implemented in IMa2.

Value	Θ_A_	Θ_PP_	Θ_FI_	m_FI->PP_	m_PP->FI_
HiPt	5.1	36.9	16.5	0.01	1.67
Mean	5.743	171.5	295.6	1.152	2.655
95%Lo	1.5	20.1	13.5	0.01	0.79
95%Hi	11.1	558.3	584.7	6.55	6.65
HPD95Lo	1.5	9.3	0		
HPD95Hi	12.9	519.3	568.5		
LRT				0.000ns	40.723***

For each parameter, the high point (HP), mean and 95% highest posterior density (95% HPD) of the marginal posterior probabilities are shown. Significant m values of the LRT are marked with asterisks:

* p<0.05;

** p<0.01;

*** p<0.001.

## Discussion

Climate during the Quaternary is characterized by the alternation between glacial and interglacial periods commonly known as the Ice Ages [1,9]. Near-shore marine benthic communities at higher latitudes would have been particularly vulnerable to the advances and retreats of continental ice sheets [84,85]. Radical glacial landscape and seascape shifts along the Magellan fjords resulted in the periodic local and temporal elimination of the associated fauna of these ecosystems [86,87] allowing the later colonization of vacant niches, as well as creating opportunities for geographical isolation and speciation [3,7,24,88]. The availability of discrete refugia may have enabled taxa to survive repeated glacial advances. Consequently, fragmentation and isolation into discrete refugia could have favored the diversification of species in the Magellan fjords [86,89]. There is a higher proportion of congeneric species south of 42°S (3.3 species per genus), than along the Central coast of Chile (1.6 species per genus). Such results suggest that the increased Patagonian diversity was generated by local radiation rather than by recent colonization from other areas as observed in New Zealand and Antarctica [55,86,89]. In this context, molecular-based genetic approaches have become fundamental to further understand and unravel the role of Quaternary glacial episodes over the distribution and demography of populations, species, and communities [1–3,5,90–93]. Mitochondrial DNA has been especially popular and represents a marker of choice in numerous phylogeographic, population genetic, and molecular taxonomy studies. Main advantages in the use these markers are: i) easy of amplification, ii) the haploid nature of the mitochondrion makes the identification of haplotypes straightforward, and iii) generally exhibit higher mutation rates compared with nuclear sequences. However, mitochondrial history does not always reflect the true history of species being studied and is often incongruent with nuclear data. Difference between the observed patterns of mitochondrial and nuclear gene variation are the consequence of introgression and incomplete lineage sorting. In spite of these caveats, and as previously stated mitochondrial DNA markers remained the marker of choice for reconstructing historical patterns of population demography, admixture, biogeography and speciation.

### Main patterns of genetic diversity and structure in *N*. *mytilina*

Levels of mtDNA genetic diversity in *N*. *mytilina* are lower than those recorded in tropical and temperate patellogastropods [94,95] and in other Patagonian nacellid limpets [20,24,25]. In fact, levels of genetic polymorphism in *N*. *mytilina* are lower than those registered in the Antarctic limpet *N*. *concinna* [96,97]. In spite of these results, main patterns of genetic diversity in *N*. *mytilina* are similar to those found in other Subantarctic nacellid limpets from New Zealand [95,98] and Marion Island [99]. Extremely low levels of genetic diversity in *N*. *mytilina* along PP could be a consequence of the drastic demographic effects during Quaternary glacial processes on the instraspecific variation of the species, as well as particular characteristics of the species, including its strong habitat dependency on macroalgae. Considering that post-glacial establishment of *N*. *mytilina* populations required the previous colonization of macroalgae (i.e. *Macrocystis*, *Lessonia*, *Gigartina*), the expansion process in *N*. *mytilina* would have been hampered or delayed. As observed in *N*. *mytilina*, molecular phylogeographic analyses in Patagonian populations of macroalgae including *Macrocystis pyrifera* (n = 144) [48] and *G*. *skottsbergii* (n = 166) [100] recorded very low levels of genetic diversity and shallow genealogies (compared to other macroalgae species). Similarly, and as observed across *N*. *mytilina*’s distribution in PP, both *Macrocystis* and *Gigartina*, exhibited the presence of a broadly distributed dominant haplotype and very low levels of genetic diversity suggesting a recent expansion processes in Patagonia. In the case of *M*. *pyrifera* the single Patagonian haplotype was also detected in several geographically distant subantarctic islands (i.e. South Georgia, Marion and Macquarie) suggesting a very recent colonization of the subantarctic region. However, in the Falkland/Malvinas Islands (n = 4), *M*. *pyrifera* individuals carried a different haplotype separated by a single substitution from the Patagonian one. In the particular case of *S*. *skottsbergi*, even when the dominant PP haplotype was also highly abundant (> 70%) in FI, no population level analyses were performed in this species in order to evaluate population differentiation [100]. Considering these results in Patagonian kelps, there is a degree of resemblence between the phylogeography of the limpet and the macroalgae where it occurs. Following this, and according to Macaya and Zuccarello [48] the high dispersal potential of kelps rafts may help to explain the absence of genetic differentiation across PP and the low genetic diversity observed in *N*. *mytilina*. At the same time, several studies have demonstrated that kelp-specialized Subantarctic invertebrates show very low levels of genetic diversity and structure along their distribution and also across geographically distant areas [101,102]. Such an hypothesis is supported by the comparative higher levels of COI genetic diversity recorded in other rocky Patagonian species of *Nacella* [20,21,24,25]. General molecular diversity indices in *N*. *mytilina* along PP (Θk = 0.89; ΘS = 1.15; ΘH = 0.90; Θπ = 1.00) would be sustained by effective population sizes (N_e_) between 73.500 and 148.000 individuals. These estimates are lower than those recorded for other Patagonian *Nacella* species [25] and by direct observations and estimates for the species [60]. Low levels of genetic diversity together with the presence of a dominant haplotype broadly distributed are consistent with the hypothesis of a recent range expansion along PP [74,103] and high levels of migration along its distribution range [3,92]. Furthermore, significant negative Tajima’s D and Fu’s F_S_ indices and the presence of L-shaped mismatch distribution in PP are the result of an excess of low frequency haplotypes commonly explained by a very recent demographic expansion process. Population expansion along PP would have occurred ~ 7.5 ka according to the sudden growth model and this estimate is consistent with analyses of other Patagonian species of *Nacella* [20,25], as well as with thermal records of warmer conditions in Patagonia. According to Hein et al. [17] the timing of the LGM extent and the onset of deglaciation occurred almost synchroneously across Patagonia. In northern areas of Patagonia the warming process started at 17.5 ka [104]. Major and rapid warming in the Strait of Magellan occurred between 14–10 ka [14,15,17,18,105]. Pollen starigraphic analyses in the Beagle Channel and Tierra del Fuego indicate that the disappearance of ice in the southern tip of South America occurred ~ 11 ka [14,15,17,106]. A postglacial molluscan faunal expansion in the Beagle Channel occurred after the full recession of glaciers around 10 ka. In fact, fossil assemblages records the diversification of different taxa, including *Nacella*, beginning at this time [107–110]. In contrast, the FI population of *N*. *mytilina* showed higher levels of genetic diversity comparable to those recorded for *N*. *magellanica* and *N*. *deaurata* in the Atlantic [20,21] and Pacific [24,25] Patagonia.

In spite of the geographical and oceanographical complexity along the southern tip of South America we recorded a single genetic unit in *N*. *mytilina* along PP from the Strait of Magellan to Cape Horn. For benthic species the primary dispersal phase is associated with the earliest life history stages (spore, egg, or larva) and therefore considerable focus is placed on the processes that influence developmental modes, type of larvae, larval development and lifespan [92,111]. Hence, the duration of these dispersal stages is expected to correlate with the dispersal ability and with the genetic structure of the organism [112–114]. Absence of genetic structure along PP has been registered in several Patagonian marine organisms with high dispersal potential including invertebrates [25,115–117] fishes [32,35,118], and macroalgae [46,48,100,119]. Regretfully, no direct information about larval duration in Patagonian species of *Nacella* exist but it is expected that their development should be similar to the Antarctic relative *N*. *concinna* [52–54]. Considering the effect of temperature on development and metabolism [120,121] it would be expected that *N*. *mytilina* larval lifespan could extend for at least six weeks. Gene flow mediated by larval dispersal and/or rafting in this kelp-dweller species may have been enhanced by oceanographic conditions in southern South America. Off southern Chile, the West Wind Drift divides in two major branches: i) the Peru or Humboldt Current that flows northward and transports subantarctic water of relatively low temperature and salinity towards lower latitudes and ii) the Cape Horn Current that flows southward around Cape Horn [122,123]. As expected under the general circulation pattern in Patagonia we recorded an absence of genetic differentiation among *N*. *mytilina* populations across Pacific Patagonia. Strong genetic differentiation between PP and FI recorded in *N*. *mytilina* supports a recent phylogeographic study in the sister-species *N*. *magellanica* [25] and in isopods of the genus *Serolis* [124] recognizing both areas as different genetic units. At the same time, FI population of *N*. *mytilina* showed higher levels of genetic diversity than PP ones that are comparable to those recorded for *N*. *magellanica* and *N*. *deaurata* populations from the Atlantic [20,21] and the Pacific [24,25] coasts of Patagonia. As stated by Leese et al. [124], while the fauna of the FI has often been accepted to share most of their faunal inventory with Patagonia, molecular analyses are indicating that shallow benthic species may in fact be strongly differentiated populations or even reproductively isolated species. Similarly, in the case of *M*. *pyrifera* even when the single Patagonian haplotype was also detected in different geographically distant subantarctic islands (i.e. South Georgia, Marion and Macquarie) it was absent in the Falkland/Malvinas Islands (n = 4). *Macrocystis pyrifera* individuals from FI carried a different haplotype separated by a single substitution from the Patagonian one. In the particular case of *S*. *skottsbergi*, even when the dominant PP haplotype is highly abundant (> 70%) in FI, no population level analyses were performed in this species in order to evaluate population differentiation [100]. Marked differences in terms of diversity, structure, and demography between PP and FI populations may be the consequence of their distinct glaciological histories during the coldest glacial periods. On the one hand, PP was almost completely covered by ice during the LGM and thus shallow marine habitats should have been severely reduced throughout this area. Accordingly, a marked decline in genetic diversity through bottleneck processes or founder effects followed by population expansion is expected [103]. Evidence of such demographic processes has been recorded in several Patagonian near-shore marine species [20,24,25,34,111–116,125,126]. In contrast, little evidence of LGM ice apart from the small cirques and short (max. 2.7 km) glacially eroded valleys are recorded in the Falkland/Malvinas Islands [14,127,128]. Moreover, an absence of widespread LGM glaciation at altitude in these islands is supported by cosmogenic isotope (^10^Be and ^26^Al) surface exposure dates [129]. Studies of Quaternary environments [130,131] provide no evidence of LGM glaciation beyond the cirques and small glaciers and there is no study showing evidence for LGM glaciers extending offshore [131]. Following this scenario, near-shore marine benthic habitats along PP would have been more hampered during LGM than in the FI, as suggested by levels of genetic diversity, haplotype genealogies, significant negative neutrality tests and L-shaped mismatch distribution. In contrast, the FI population showed deviation from the mutation-drift equilibrium model and exhibited multimodal mismatch distribution, in conformity with the expectation for more stable populations. Major differences between PP and FI in terms of habitat availability has been recognized in comparative biodiversity analyses [132].

### Source-sink model as consequence of asymmetrical gene flow

Traditional genetic models of postglacial recolonization and refugia include the prediction that formerly ice-covered areas should exhibit low levels of genetic diversity and a small number of haplotypes dominating large areas [1]. Simultaneously, recolonized areas should also exhibit low divergence among haplotypes and lower levels of genetic structure than refugial ones. Following our results, and considering this general Quaternary genetic model [1–3], FI could be then considered as a glacial refugium for *N*. *mytilina*. In fact, these islands have been previously proposed as refugial areas for several plant species during the LGM [133] and as an important area for conservation [134]. MtDNA data in *N*. *concinna*, as well as in its sister-species *N*. *magellanica*, support the resiliency of some near-shore marine benthic invertebrates in FI during the LGM. At the same time, our migration analyses in *N*. *mytilina* do not support an scenario of posterior colonization from FI to PP.

An asymmetrical dispersal pattern from PP to FI recorded in *N*. *mytilina* is strongly supported by the general oceanographic circulation in the study area. After rounding the southern tip of South America, the Cape Horn Current is divided into two minor branches. The first one flows eastward to the South Georgia [122,123] while the second one flows northwards on both sides of the Falkland/Malvinas Islands up north to Rio de la Plata Province (30°S) following the continental shelf margin [135,136]. As previously proposed for another Patagonian nacellid limpet [25], FI seems to represent a sink area, as well as a secondary contact zone where endemic haplotypes that survived the LGM are mixed together with recently arrived PP ones. Future studies in Patagonian near-shore marine benthic invertebrates will require to integrate mtDNA/nucDNA genes together with fast-evolving markers (microsatellites, RADseq, SNPs) to further understand the role of the Quaternary cycles over the current patterns of genetic diversity, structure and connectivity. At the same time, it is also required to expand the sampling effort toward areas of difficult access that have been neglected in previous studies.

## Main Conclusions

Molecular-based phylogeographic analyses in *N*. *mytilina* indicate that historical and contemporary processes, as well as life-history traits played a major role in the current pattern of genetic diversity, structure, and connectivity. This study corroborates previous analyses in the genus showing the important role of oceanography in explaining the asymmetry in gene flow across the southern tip of South America. Even in the presence of gene flow from PP to FI, the migration between both areas is not enough to homogenize populations and they constitute different genetic units. According to the general pattern of genetic diversity, neutrality tests and demographic inference analyses, *N*. *mytilina* populations in FI are older than those from PP. However, haplotype identities and gene flow analyses indicate that FI did not participate in the postglacial recolonization of PP. In this context, more studies in other shallow marine benthic organisms are required to determine if the pattern recorded in two species of *Nacella* constitute a general rule of the Quaternary biogeography of southern South America.
